# Editorial: Innovations and impacts of antimicrobial stewardship in primary care and proximity settings

**DOI:** 10.3389/frabi.2026.1995353

**Published:** 2026-10-01

**Authors:** Elmien Bronkhorst

**Affiliations:** Department of Clinical Pharmacy, Sefako Makgatho Health Sciences University, Pretoria, South Africa

**Keywords:** animal antimicrobial use, antimicrobial resistance, antimicrobial stewardship, community settings, primary care, self-care

Antimicrobial resistance (AMR) poses a significant threat to global health and remains one of the most pressing public health challenges of the twenty-first century. Innovative antimicrobial stewardship (AMS) approaches are needed to mitigate its impact, expanding into diverse community healthcare settings. This includes primary care settings and proximity, where most human and animal antimicrobial exposure occurs. Individualized, community-based approaches to stewardship, extending beyond conventional prescribing in hospital-settings, are recognized. This includes public engagement, education on self-care, inclusion of veterinary sectors and broader societal determinants of AMR. Technological advancements and policy implementations highlight the continuous evolution of stewardship measures, underscoring the need for ongoing research, interdisciplinary collaboration, and adaptive frameworks to address emerging challenges.

The goal of this Research Topic is to explore the latest innovations and impacts of antimicrobial stewardship in primary care and proximity settings, and identify barriers to stewardship in these settings. The papers in this collection provide valuable insights into the evolving landscape of AMS across different target populations and settings. It seeks to highlight successful strategies and interventions and the overall impact on patient outcomes and public health. Furthermore, they describe ways to overcome challenges and barriers to implementation. By sharing this collection, we hope to provide a deeper understanding of effective AMS practices, encourage the adoption of best practices, and identify areas requiring further investigation. Collectively, they illustrate the importance of adopting integrated, multisectoral strategies to address AMR.

An innovation discussed by Kiggundu et al. is the integration of self-care strategies into AMR containment efforts. Their review broadens the traditional stewardship paradigm, shifting from healthcare providers to positioning individuals as active participants in infection prevention and antimicrobial decision-making. Through AMR education, self-care interventions have the potential to improve health literacy, enable earlier detection of infections, and reduce unnecessary healthcare utilization. This was facilitated by rapid diagnostic self-testing, guided self-treatment, self-monitoring and supported self-management. Importantly, digital health technologies and community health systems emerge as key enablers of responsible self-care. However, the authors caution that self-care is not risk-free and benefits may be undermined by limited access to reliable information, diagnostic uncertainty and the potential for inappropriate antibiotic use. Their work highlights an important future direction for stewardship: empowering communities while ensuring adequate safeguards, regulation, education, and professional support.

The importance of sustained public engagement is further demonstrated by Bajčetic et al., who describe one of Europe’s most successful national antibiotic awareness initiatives. Serbia’s multisectoral educational campaign “Careful with Antibiotics”, combined public education, professional training, guideline development, and regulatory reforms. The results show one of the most substantial and sustained reduction in systemic antibiotic prescriptions in Europe. Encouraging is the reduction in antibiotic prescribing for common respiratory tract conditions that have historically driven inappropriate antibiotic use. This work demonstrates that AMS is most effective when it extends beyond clinical practice into public communication, professional education, and health policy. The Serbian experience provides a compelling example for countries seeking scalable and sustainable stewardship interventions in primary care.

The challenges of stewardship beyond human healthcare are highlighted by Mugyabuso et al., whose study emphasizes the importance of a One Health perspective. Their findings reveal low awareness of antimicrobial use, residues, and resistance among cattle keepers, accompanied by poor stewardship practices and significant regulatory weaknesses. The widespread availability of non-prescription antimicrobials, reliance on empirical treatment decisions, and limited AMR knowledge among some animal health stakeholders represent important threats to stewardship efforts. The study identifies education and awareness as modifiable factors strongly associated with responsible antimicrobial attitudes. These findings highlight that stewardship in proximity settings must encompass agricultural and veterinary sectors. Efforts to improve prescribing practices in human healthcare may achieve only limited success if parallel challenges in animal health remain unaddressed.

While the first three contributions focus on strengthening stewardship interventions, Amábile-Cuevas presents an important and thought-provoking challenge to conventional AMS assumptions. The author argues that the relationship between antibiotic consumption and resistance is more complex than traditionally acknowledged. Socioeconomic, environmental, and biological factors may influence resistance patterns as strongly as, or more than clinical antibiotic use. This perspective does not dismiss the value of stewardship but rather encourages a reassessment of its objectives. Stewardship programs undoubtedly improve patient safety, reduce unnecessary antimicrobial exposure, and lower healthcare costs. However, expecting AMS alone to reverse resistance trends may be unrealistic considering broader drivers such as agricultural antimicrobial use, urbanization, sanitation challenges, and the dissemination of mobile genetic elements. This argument reinforces the need for stewardship efforts to be embedded within wider health systems and incorporate societal approaches.

Taken together, the contributions in this collection highlight several recurring themes. First, AMS is increasingly moving beyond hospitals into communities, households, pharmacies, and agricultural settings. Second, effective stewardship requires engagement of multiple stakeholders, including patients, caregivers, pharmacists, veterinarians, policymakers, and public health practitioners. Third, education remains a cornerstone of stewardship, whether directed at self-carers, parents, healthcare professionals, or livestock keepers. Finally, the complexity of AMR demands that stewardship strategies evolve from narrowly focused prescribing interventions toward integrated approaches that address behavioral, environmental, regulatory, and socioeconomic determinants.

The future of AMS in primary care and proximity settings depends on our ability to balance innovation with evidence-based implementation. Self-care technologies, digital health tools, public awareness campaigns, and One Health interventions offer promising opportunities to improve antimicrobial use. Simultaneously, stewardship programs must remain cognizant of their limitations and continuously adapting to the multifactorial nature of AMR. This collection demonstrate that meaningful progress is achievable when stewardship is viewed not merely as a clinical intervention, but as a shared societal responsibility.

As AMR continues to threaten global health gains, these contributions reaffirm that effective stewardship must be collaborative, context-specific, and inclusive. By bringing AMS closer to the communities, primary care and proximity settings are poised to become critical frontiers in the global response to AMR.

